# The Evidence for Association of *ATP2B2* Polymorphisms with Autism in Chinese Han Population

**DOI:** 10.1371/journal.pone.0061021

**Published:** 2013-04-19

**Authors:** Wen Yang, Jing Liu, Fanfan Zheng, Meixiang Jia, Linnan Zhao, Tianlan Lu, Yanyan Ruan, Jishui Zhang, Weihua Yue, Dai Zhang, Lifang Wang

**Affiliations:** 1 Key Laboratory of Mental Health, Ministry of Health (Peking University), Beijing, People’s Republic of China; 2 Institute of Mental Health, Peking University, Beijing, People’s Republic of China; 3 Beijing Children’s Hospital Affiliated to Capital University of Medical Sciences, Beijing, People’s Republic of China; 4 Peking-Tsinghua Center for Life Sciences, Beijing, People’s Republic of China; Baylor College of Medicine, United States of America

## Abstract

**Background:**

Autism is a neurodevelopmental disorder with a high estimated heritability. *ATP2B2*, located on human chromosome 3p25.3, encodes the plasma membrane calcium-transporting ATPase 2 which extrudes Ca^2+^ from cytosol into extracellular space. Recent studies reported association between *ATP2B2* and autism in samples from Autism Genetic Resource Exchange (AGRE) and Italy. In this study, we investigated whether *ATP2B2* polymorphisms were associated with autism in Chinese Han population.

**Methods:**

We performed a family based association study between five SNPs (rs35678 in exon, rs241509, rs3774180, rs3774179, and rs2278556 in introns) in *ATP2B2* and autism in 427 autism trios of Han Chinese descent. All SNPs were genotyped using the Sequenom genotyping platform. The family-based association test (FBAT) program was used to perform association test for SNPs and haplotype analyses.

**Results:**

This study demonstrated a preferential transmission of T allele of rs3774179 to affected offsprings under an additive model (T>C, Z = 2.482, *p* = 0.013). While C allele of rs3774179 showed an undertransmission from parents to affected children under an additive and a dominant model, respectively (Z = −2.482, *p* = 0.013; Z = −2.591, *p* = 0.0096). Haplotype analyses revealed that three haplotypes were significantly associated with autism. The haplotype C-C (rs3774180–rs3774179) showed a significant undertransmission from parents to affected offsprings both in specific and global haplotype FBAT (Z = −2.037, *p* = 0.042; Global *p* = 0.03). As for the haplotype constructed by rs3774179 and rs2278556, C-A might be a protective haplotype (Z = −2.206, *p* = 0.027; Global *p* = 0.04), while T-A demonstrated an excess transmission from parents to affected offsprings (Z = 2.143, *p* = 0.032). These results were still significant after using the permutation method to obtain empirical *p* values.

**Conclusions:**

Our research suggested that *ATP2B2* might play a role in the etiology of autism in Chinese Han population.

## Introduction

Autism is a neurodevelopmental disorder characterized by severe impairment in reciprocal social interaction and communication skills, and the presence of stereotyped behaviors and interests [1]. Previous researches reported that autism spectrum disorders (ASD) occurred in 1 out of 150 individuals [2]. Several lines of evidence suggested that strong genetic components were involved in the susceptibility to ASD [3]–[5]. Twin studies indicated that the estimated heritability of autism was nearly 90%. However, autism is a common disorder with multiple genetic causes. Recently, genome wide association studies (GWA) identified susceptibility common genetic variants (i.e. rs4307059 in 5p14.1 [6], rs10513025 in 5p15.2 [7], rs4141463 in the intron of *MACROD2* [8]) for ASD which support the “common disease, common variant” hypothesis of this complex disease [9] . However, no definitely replicated results were reported across literatures until now. Common variants dredged from simple GWAS analysis could not explain a substantial fraction of the heritability of ASD [10]. It seems that a myriad of common variations of very small effect impacts ASD liability [11] . Rare variants such as mutations and/or copy number variants (CNV) might attributable to the “missing heritability” [12] . These lines of evidence support the other hypothesis “common disease, rare variant”, which proposes that many rare variants and/or structural variants (i.e. CNV) might contribute to the risk of common disease [13]–[15] . Candidate gene approaches are also useful to detect the common and/or rare variants associated with ASD.

*ATP2B2* (ATPase, calcium-transporting, plasma membrane 2) encodes plasma membrane calcium-transporting ATPase isoform 2 (PMCA2). PMCA2 belongs to the family of P-type primary ion transport ATPases which remove bivalent calcium ions from eukaryotic cells against very large concentration gradients and play a critical role in intracellular calcium homeostasis. Intracellular calcium signaling is important for the development of specific synaptic connections. Disruptions of intracellular calcium homeostasis underlie a host of emerging diseases such as seizures and autism [16]. Evidence from biochemical and genetic studies of the mitochondrial aspartate/glutamate carrier AGC1 indicated that AGC and altered Ca^2+^ homeostasis play a key role in the cascade of signaling events leading to autism [17]. Therefore, understanding the role of calcium in setting up brain networks may help identifying the causes of diseases such as autism.

Furthermore, *ATP2B2* is expressed mainly in cerebellum, and also expressed in cerebral cortex, olfactory bulb, and hippocampal formation [18]–[22]. Previous researches have demonstrated that absence of ATP2B2 led to defects of both the vestibular and auditory systems [23]–[27]. Moreover, several studies suggested that *ATP2B2* plays an important role in synaptic signaling and functional cerebellar output [28]–[30]. And *ATP2B2* had a strong influence on development and maintenance of the cerebellar function [31].

*ATP2B2* is located in human chromosome region 3p25.3. Several studies identified region of suggestive linkage with autism on chromosome 3p25 [32]–[34]. There might reside some susceptibility genes for autism. Previous GWAS did not detect the association between *ATP2B2* and autism [6]–[8] . Recently, Carayol J, et al. performed a family-based association study between *ATP2B2* and autism. They firstly provided the evidence for a susceptibility gene *ATP2B2* for autism [35]. Then this result was replicated in another research in Italy [36]. These researches indicated that *ATP2B2* might be a predisposing gene for autism.

The biological functions of ATP2B2 and the positive results of previous association made *ATP2B2* become an attractive candidate gene for autism. Considering the genetic heterogeneity in different ethnicities, we performed a family-based association study to investigate the association between *ATP2B2* and autism in Chinese Han population.

## Materials and Methods

### Ethics Statement

This research was approved by the Ethics Committee of Institute of Mental Health, Peking University. All subjects provided written informed consent, and informed written consent for children was obtained from their biological parents (the children’s legal guardians).

### Subjects

We performed a family based association study in 427 autism trios of Han Chinese descent (1281 individuals, including probands and their biological parents). All subjects were recruited at the Institute of Mental Health, Peking University, China. Of the 427 autistic children, 403 were male and 24 were female. The mean age of the children at the time of testing was 6.3 years old (ranging from 2 to 16.7 years). These children were diagnosed with autism by two senior psychiatrists, and all children met the DSM-IV criteria for autism. Besides, these autistic children were assessed using Autism Behavior Checklist (ABC) [37] and Childhood Autism Rating Scale (CARS) [38]. Children who had scores ≥53 on ABC and ≥35 on CARS were included in the present study. The demographic data were listed in Table S1 . Children with Asperger’s syndrome, Rett syndrome, phenylketonuria, fragile X syndrome, tuberous sclerosis and a previously identified chromosomal abnormality were excluded. Children with pervasive developmental disorder not otherwise specified (PDD-NOS) which also called atypical autism were not included in this study. As for PDD-NOS, the criteria for autistic disorder were not met, for instance because of late age of onset, atypical symptomatology, or subthreshold symptomatology, or all of these.

### SNP Selection and Genotyping

Genomic DNA was extracted from blood samples using the QIAamp DNA Blood Mini Kit. The SNPs information was obtained from dbSNP database (http://www.ncbi.nlm.nih.gov/SNP/) and HapMap phaseII genotype Chinese Han in Beijing (CHB) dataset (http://www.hapmap.org). Previous studies had reported that four SNPs (rs35678 in exon, rs241509, rs3774180, and rs2278556 in introns) were associated with autism [35], [36]. To explore whether these results could be replicated in Chinese Han population, we selected the above-mentioned four SNPs (rs35678 (NC_000003.11:g.10379923C>T) in exon, rs241509 (NC_000003.11:g.10387059C>A), rs3774180 (NC_000003.11:g.10396988T>C), and rs2278556 (NC_000003.11:g.10402103G>A) in introns) and another SNP rs3774179 (NC_000003.11:g.10397069T>C) which is close to rs3774180 in the present association study. The minor allele frequencies (MAF) of these five SNPs in Chinese Han population were more than 0.05.

All SNPs were genotyped using the iPLEX Assay on the Sequenom MassARRAY platform (Sequenom Inc, San Diego, California), which uses the matrix-assisted laser desorption/ionization time-of-flight (MALDI-TOF) mass spectrometry primer extension assay [39], [40]. Primers were designed by Sequenom online service (http://www.sequenom.com). Information of primers was listed in Table 1. We used iPLEX genotyping assay, which has increased plexing efficiency and flexibility for the MassARRAY system through single base primer extension with mass-modified terminators [41]. Polymerase chain reaction (PCR) was performed according to the standard protocol. Spectra were analyzed using MassARRAY Typer, version 4.0 software (Sequenom). Quality control was performed by excluding individual SNPs or samples with genotype call rates less than 95% and SNP assays with poor-quality spectra/cluster plots.

**Table 1:** The information of SNPs in *ATP2B2* and primers of PCR and unextended primers for Sequenom SNP genotyping.

SNP	Location	2nd-PCR	1st-PCR	UEP_SEQ
rs35678	Exon	ACGTTGGATGAACGAGGACGTGGAGGAGAT	ACGTTGGATGTGTCTGGATCCGATTCAGGC	GTGTAGGAGATCGACCACGC
rs241509	Intron	ACGTTGGATGCGTGATTGTGGCCTTCACAG	ACGTTGGATGTCCCCCAGGCTTTGGTGTG	ACTTCATCACGCAGGTGGGTCC
rs3774180	Intron	ACGTTGGATGAAACTGAGGCCATCTGTGAG	ACGTTGGATGACTCAAGTGACACAGCAGAC	GTGGCAGGGGCAGAA
rs3774179	Intron	ACGTTGGATGTTCCTTCAGTCAGGGCATCC	ACGTTGGATGGTGAGACAGTTTATGACTCC	GGGGATCCTGTTGGCTCACCCCTGGG
rs2278556	Intron	ACGTTGGATGAAAATCTGAGGCAAGTACGG	ACGTTGGATGGTTACGTGCCTATCATCCAG	ACGGCATAATGTTAAGATG

SNP, single nucleotide polymorphism; PCR, polymerase chain reaction; UEP, unextended primer.

To confirm the genotype results by Sequenom, all these five SNPs were regenotyped in 10% of the samples. Direct DNA sequencing was used for analyzing rs35678, rs3774180, rs3774179 and rs2278556, which do not have natural restriction sites. The SNP rs241509 was analyzed by polymerase chain reaction-restriction fragment length polymorphism (PCR-RFLP) analysis. Details for PCR-RFLP and direct sequencing were described in the Text S1.The information of primers and PCR-RFLP analysis were listed in Table S2. Raw data are available upon request. All DNA sequencing data have been deposited in GenBank. The genotyping concordance rate for Sequenom and direct sequencing was more than 99%, and that was 96% for Sequenom and PCR-RFLP.

### Statistical Analysis

Prior to analysis, Mendelian inconsistencies were checked using the PEDCHECK program, version 1.1 [42]. The Hardy-Weinberg equilibrium for genotype frequency distributions was tested using the Chi-square goodness-of-fit test. We used the Haploview 4.1 (http://www.broad.mit.edu/mpg/haploview/) to calculate the pair-wise *D′* values for SNPs and construct haplotypes. SNP pairs were considered to be in strong linkage disequilibrium (LD) if *D*′>0.8. The family-based association test (FBAT) program version 2.0.4 (beta1) (http://www.biostat.harvard.edu/~fbat/default.html) was used to perform association test for SNPs under additive model and dominant model [43]. Moreover, FBAT program provided estimated haplotype frequencies and pairwise LD between the specific markers. The individual haplotype tests were conducted under “biallelic” mode in haplotype based association test (HBAT). Meanwhile, the global haplotype tests of association were performed under “multiallelic” mode in HBAT. The significance level for all statistical tests was two-tailed (*p*<0.05).

The power of sample size for association tests was evaluated using Quanto software version 1.2.4 (http://hydra.usc.edu/gxe/) [44] . The sample size of 427 trios had approximately 82% power to detect risk allele frequencies ranging from 0.191 to 0.451, assuming a relative risk of 1.4, the prevalence of autism of 0.006, with additive model.

Correction for multiple testing was conducted using Bonferroni correction (α/N = 0.05/5 = 0.01). However, Bonferroni correction is too conservative to detect small or moderate effects. Considering the inter-correlation of the SNPs, SNPSpD (http://genepi.qimr.edu.au/general/daleN/SNPSpD) program was used as a simple correction for multiple testing of SNPs in LD with each other [45] . SNPSpD, on the basis of the spectral decomposition (SpD) of matrices of pairwise LD between SNPs, automatically removed redundant SNPs (i.e., SNPs in completed LD (r^2^ = 1) with another SNP) [46] . In our data, the effective number of independent marker loci calculated by SNPSpD was 3. The calculated significance threshold required to keep type I error rate at 5% was 0.01695.

## Results

Five SNPs (rs35678, rs241509, rs3774180, rs3774179, and rs2278556) in *ATP2B2* were genotyped in 427 autism trios (1281 individuals). The call rates of individual SNPs and samples were more than 98%. All SNP assays were with good quality cluster plots.

All of these five SNPs were polymorphic with minor allele frequencies more than 5%. Therefore, these SNPs were used as genetic markers in this study. None of the genotype distributions of these SNPs in parents significantly deviated from Hardy-Weinberg equilibrium (*p*>0.05). The allele frequencies, genotype frequencies, and *p* value for Hardy-Weinberg equilibrium of these five SNPs in children affected with autism and parents were shown in Table S3.

Univariate (single marker) test using FBAT demonstrated that T alleles of rs3774179 showed a preferential transmission from parents to children affected with autism (T>C, Z = 2.482, *p* = 0.013, under an additive model). While C allele of rs3774179 showed a significant undertransmission from parents to affected children under an additive model and a dominant model, respectively (Z = −2.482, *p* = 0.013; Z = −2.591, *p* = 0.0096). Allele frequencies and the association results for single SNP analyses were shown in Table 2. The results were similar in trios with male probands (data not shown).

**Table 2:** Results of association between 5 SNPs in *ATP2B2* and autism and haplotype analyses in 427 trios of Han Chinese descent.

Marker	Allele	Afreq	Additive model					Dominant model				
			Families	S- E(S)	Var(S)	Z	*p*	Families	S- E(S)	Var(S)	Z	*p*
rs35678	C	0.429	287	−9	94.5	−0.926	0.355	216	−4.8	48.3	−0.683	0.494
	T	0.571	287	9	94.5	0.926		162	4.2	34.8	0.720	0.471
rs241509	A	0.549	302	−1.5	102.8	−0.148	0.882	181	7.2	38.4	1.169	0.242
	C	0.451	302	1.5	102.8	0.148		230	8.7	50.7	1.229	0.219
rs3774180	C	0.551	296	−6.5	98.3	−0.656	0.512	173	−1.8	37.2	−0.287	0.774
	T	0.449	296	6.5	98.3	0.656		220	4.7	48.9	0.679	0.497
rs3774179	C	0.191	211	−19.5	61.8	−2.482	**0.013**	202	−18	48.3	−2.591	**0.0096**
	T	0.809	211	19.5	61.8	2.482		45	1.5	9.0	0.500	0.617
rs2278556	A	0.595	305	1.5	95.3	0.154	0.878	149	−5.5	32.5	−0.965	0.335
	G	0.405	305	−1.5	95.3	−0.154		232	−7.0	53.3	−0.959	0.337
**Marker**	**Haplotype**	**Afreq**	**Families**	**S- E(S)**	**Var(S)**	**Z**	***p***	**Global**		***p*** **_permutation_** a	***p*** **_permutation_** **(whole marker)**	
								**χ^2^**	***p***			
rs3774180–rs3774179												
	C-C	0.185	198.6	−15.8	60.3	−2.037	**0.042**	8.95	**0.030**	**0.042**	**0.040**	
	C-T	0.362	271.1	9.3	86.9	1.000	0.317			0.316		
	T-T	0.447	286.5	9.7	96.5	0.985	0.325			0.320		
rs3774179–rs2278556												
	C-A	0.183	201.8	−17.0	59.4	−2.206	**0.027**	8.32	**0.040**	**0.027**	**0.035**	
	T-A	0.410	279.1	20.5	91.5	2.143	**0.032**			**0.032**		
	T-G	0.400	294.5	−1	92.8	−0.104	0.917			0.905		
rs3774180–rs3774179–rs2278556												
	C-C-A	0.182	197.2	−16.1	58.2	−2.117	**0.034**	9.14	0.104	**0.033**	0.202^b^	
	C-T-A	0.337	246.1	13.1	80.6	1.464	0.143			0.149		
	T-T-G	0.371	260.4	3.6	88.3	0.375	0.708			0.686		
	T-T-A	0.074	102.8	6.1	28.0	1.155	0.248			0.249		

Afreq, allele frequency; Families, number of informative families; S, test statistics for the observed number of transmitted alleles; E(S), expected value of S under the null hypothesis (i.e., no linkage and no association).

a The number of permutationS is 10,000;

b whole marker permutation test using minimal *p* (when only one haplotype was significant).

**Figure 1:**
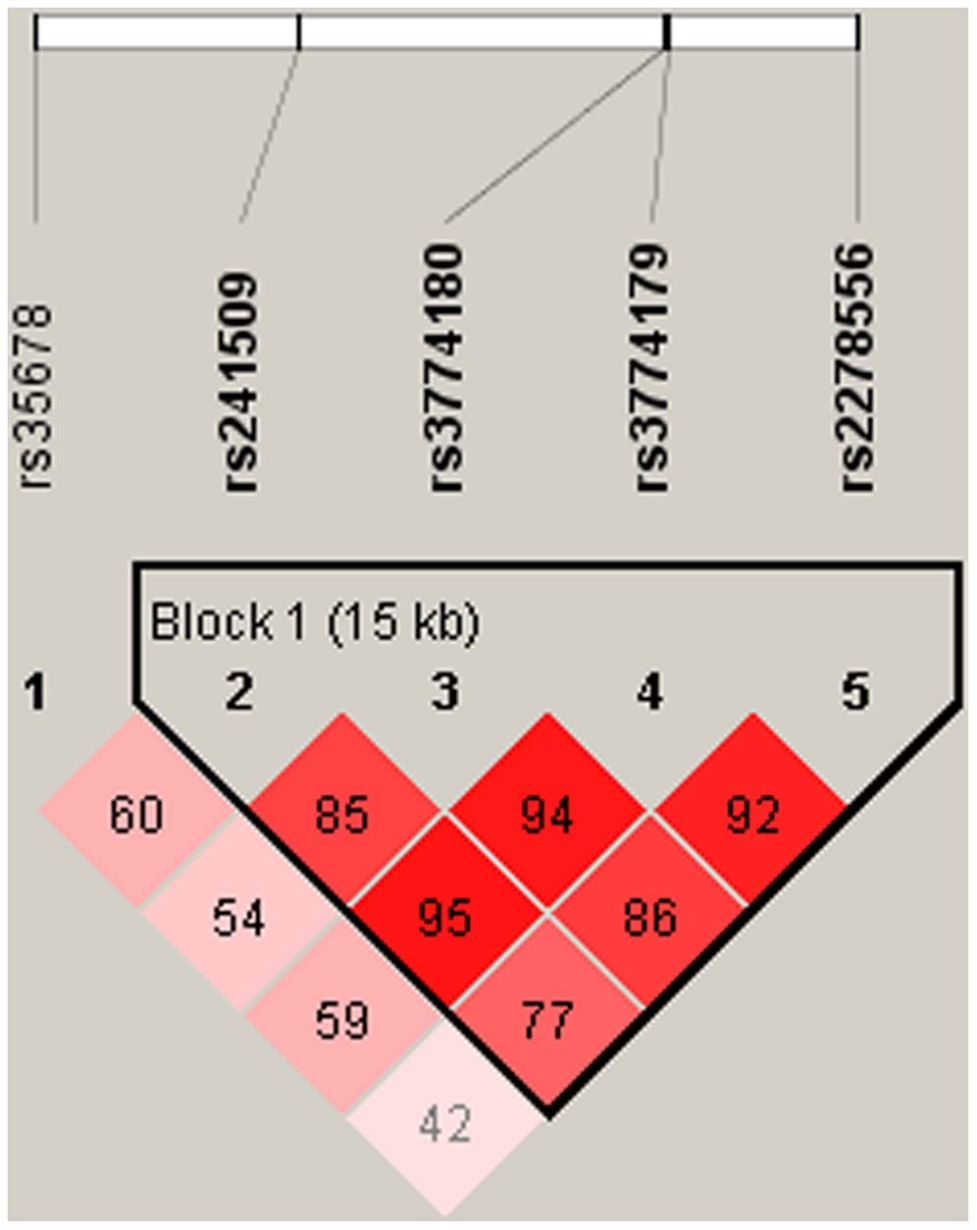
Linkage disequilibrium (LD) block constructed from 5 SNPs in *ATP2B2*. Markers with Linkage disequilibrium (LD) (*D’*<1 and LOD>2) are shown in red through pink (color intensity decreases with decreasing *D’* value). *D’* value shown within each square represents a pairwise LD relationship between the two polymorphisms. *D* prime values of 1.0 are never shown (the box is empty). The LD plot was generated with the Haploview program.

The LD patterns of the SNPs were measured with *D* primer value using Haploview. Four SNPs (rs241509, rs3774180, rs3774179 and rs2278556) were identified in one LD block with *D*’ value range from 0.77 to 0.95 (Figure 1).

Bonferroni correction for multiple testing (5 tests) resulted in a significance level of *p* = 0.01, and only the result for rs3774179 under a dominant model remained significant. Considering Bonferroni correction is too conservative, SNPSpD approach was used as a simple correction for multiple testing of SNPs in LD with each other. The calculated significance threshold required to keep type I error rate at 5% was 0.01695. Therefore, rs3774179 was significantly associated with autism both under an additive model (*p* = 0.013) and a dominant model (*p* = 0.0096) after correction of multiple comparisons.

To determine whether any specific haplotypes would confer a higher risk for autism, the specific and global haplotype association tests were performed. Haplotypes were constructed by neighboring SNPs using a sliding window approach. Three haplotypes were significantly associated with autism (Table 2). The haplotype C-C (rs3774180–rs3774179) showed a significant undertransmission from parents to affected offsprings both in specific and global haplotype FBAT (Z = −2.037, *p* = 0.042; Global *p* = 0.03). As for the haplotype constructed by rs3774179 and rs2278556, C-A might be a protective haplotype (Z = −2.206, *p* = 0.027; Global *p* = 0.04), while T-A demonstrated an excess transmission from parents to affected offsprings (Z = 2.143, *p* = 0.032). These results were still significant after using the permutation method to obtain empirical *p* values (Table 2). The haplotype C-C-A (rs3774180–rs3774179–rs2278556) revealed an undertransmission from parents to children affected with autism in the specific HBAT (*Z* = −2.117, *p = *0.034, *p_permutation_ = *0.033). However, the global *p* value of haplotypes encompassing rs3774180–rs3774179–rs2278556 did not show significant (*p* = 0.104). Although rs3774179 might contribute to the association of these haplotypes, haplotypes could capture the combined effects of tightly linked cis-acting causal variants [47] . Other results of haplotype analyses for haplotype constructed by the remaining SNPs were shown in Table S4. When performed association study in trios with male probands, the results had similar trend (data not shown).

## Discussion

In this present study, we investigated the association between *ATP2B2* polymorphisms and autism in Chinese Han population. The results of our research indicated that rs3774179 in *ATP2B2* was significantly associated with autism (*p* = 0.013, under an additive model; *p* = 0.0096, under a dominant model). Haplotype analyses showed that three haplotypes were significantly associated with autism, all of which including rs3774179.

Two previous family-based association studies explored the association between *ATP2B2* and ASD. One reported that SNPs (rs241509, rs3774180 and rs2278556) represented positive association with autism in their exploratory AGRE samples and in the replication samples from Italy [35]. Moreover, they did meta-analysis of the exploratory and replication results in the recessive model after correction for multiple testing, observing strongly significant association between rs35678 (*p* = 0.0023), rs241509 (*p* = 0.00026), rs3774180 (*p* = 0.000018), rs2278556 (*p* = 0.00013) and autism [35]. In the other research based on Italian ASD families, results showed that homozygotes of rs35678 (individuals with genotype CT were at lower risk compared with CC or TT individuals, *p* = 0.0013) were associated with ASD [36]. However, these results were not replicated in our samples in Chinese Han population. We compared the allele frequencies of these SNPs in different populations using the data downloaded from Hapmap data (Table S5). For rs35678, rs241509, rs3774180, and rs3774179, the allele frequencies in Chinese Han in Beijing, China (CHB) population were similar to those in Utah residents with Northern and Western European ancestry from the CEPH collection (CEU) population. However, the allele frequency of rs2278556 was 0.440 for G allele in CHB population while it was 0.624 in CEU population.

We detected that rs3774179 which is near to rs3774180 was significantly associated with autism. Previous research by Carayol J, et al. reported that rs3774180 was associated with autism [35]. However, they did not perform association test between rs3774179 and autism. The difference between the results in our research and previous researches might have a few reasons. One was the genetic heterogeneity in different ethnicities. For example, the allele frequency of rs2278556 is different between CHB and CEU populations. Second, the rs3774179 which was associated with autism in our research is near to rs3774180. As only a few tagSNPs were chosen to genotype according to the patterns of LD, markers tested for association must either be the causal allele or highly correlated with the causal allele [48], [49]. Another reason was that children affected with Asperger’s syndrome and PDD-NOS were excluded in our research. It was reported that the *p* values may decreased (i.e. the association became stronger) when the phenotype definition of autism was relaxed to a “spectrum” definition [35].

ATP2B2 is expressed at high level in cerebellum and abundant throughout the cerebral cortex and hippocampus, particularly clustering in the dendrites and spines of Purkinje cells in cerebellum [20], [22]. Several previous researches suggested that ATP2B2 plays an important role in modulating Ca^2+^ signaling at the synapse, glutamate receptor expression, and survival of Purkinje cells [30], [50]–[52]. Neural development is associated with intracellular Ca^2+^ levels which can activate signaling pathways to regulate neuronal survival, differentiation, migration, and synaptogenesis [53], [54]. Any defects of these developmental stages may lead to neuroanatomical abnormalities, decreases of the size and density of neurons, alterations in neural connectivity. For example, dysfunction of ATP2B2 might lead to decreased expression of metabotropic glutamate receptor and aberrant signaling in Purkinje neurons, resulting in functional deficits of cerebellum [55], which usually can be detected in autism [56]–[59]. Direct functional evidence demonstrated the influence of presynaptic ATP2B2-mediated Ca^2+^ extrusion for short-term plasticity at cerebellar parallel fiber to Purkinje neuron synapses. It indicated that ATP2B2 had a strong influence on the development and maintenance of cerebellar function [31]. Recent researches indicated that in *Atp2b2* ^+/−^ mouse model, the dysregulation of Ca^2+^ signaling and the perturbations in glutamate receptor signaling complexes were paralleled by delayed Purkinje cells loss, and presumed that these mechanisms might be relevant to Purkinje cells dysfunction and loss in autistic patients [51], [60], [61].

The present study had a few limitations. First, the aim of this study was to replicate previous positive results of association between *ATP2B2* and autism. Therefore, our research did not explore the association between SNPs in promoter region and 3′ untranslated region in *ATP2B2* with autism. Second, rare variants were not investigated in this study. Mutation screening of *ATP2B2* in children with autism could be performed in further researches. Third, the sample size of our research is needed to be expanded.

In conclusion, our present results provided evidence that *ATP2B2* might be relevant to the etiology of autism. Further association study in larger samples and functional researches of *ATP2B2* are needed.

## Supporting Information

Table S1 **The demographic data of subjects affected with autism.**
(DOC)

Table S2 **The information of primers and PCR-RFLP analysis of five SNPs in** ***ATP2B2*** **.**
(DOC)

Table S3 **Information of 5 SNPs in** ***ATP2B2*** **and the genotype frequencies in 427 autism trios of Chinese Han descent.**
(DOC)

Table S4 **Estimated haplotype frequencies and results of haplotype association analyses of** ***ATP2B2*** **.**
(DOC)

Table S5 **Comparison of the allele frequencies for the five SNPs in CHB and CEU from HapMap data.**
(DOC)

Text S1 **Details for PCR-RFLP (polymerase chain reaction-restriction fragment length polymorphism) and direct sequencing.**
(DOC)
