# Supplementary material for: The Evidence for Association of ATP2B2 Polymorphisms with Autism in Chinese Han Population
Source: PLoS One. 2013 Apr 19;8(4):e61021. doi: 10.1371/journal.pone.0061021 (PMC3631200; doi:10.1371/journal.pone.0061021)
Supplement: Table S1 — The demographic data of subjects affected with autism. (DOC) [file pone.0061021.s001.doc]

**Table S1. The demographic data of subjects** **affected with autism.**

|  | Mean±SD |
| --- | --- |
| Age | 6.3^a^ |
| CARS | 42.57±4.12 |
| ABC | 64.05±5.45 |

SD, Standard Deviation; CARS, Childhood Autism Rating Scale; ABC, Autism Behavior Checklist; ^a^ median.
