# Supplementary material for: The Evidence for Association of ATP2B2 Polymorphisms with Autism in Chinese Han Population
Source: PLoS One. 2013 Apr 19;8(4):e61021. doi: 10.1371/journal.pone.0061021 (PMC3631200; doi:10.1371/journal.pone.0061021)
Supplement: Table S2 — The information of primers and PCR-RFLP analysis of five SNPs in ATP2B2 . (DOC) [file pone.0061021.s002.doc]

**Table S2. The information of primers and PCR-RFLP analysis of five SNPs in *ATP2B2***.

| SNP | Position | Primer sequence (5´→3´) | Product (bp) | RFLP | Allele (bp) | |
| --- | --- | --- | --- | --- | --- | --- |
| rs35678^a^ | Exon | F: 5´-GAGCGACAGTCCCCTCACTCAC-3´  R: 5´-AGAGCGGCCCCCACTTTACAG-3´ | 925 | - | - | - |
| rs241509 | Intron | F: 5´-GGCCAGTGCCTGCACCTGAG-3´  R: 5´-GGCCAGGGTGAGGCTTCCTGA-3´ | 575 | PpuMI | A: 174/401 | C: 575 |
| rs3774180^a^ | Intron | F: 5´-CCCACTCGGAGGTGCTGAATC-3´  R: 5´-TTGCCGCTAACGAGCCACTTC-3´ | 458 | - | - | - |
| rs3774179^a^ | Intron | F: 5´-CCCACTCGGAGGTGCTGAATC-3´  R: 5´-TTGCCGCTAACGAGCCACTTC-3´ | 458 | - | - | - |
| rs2278556^a^ | Intron | F: 5´-CACGGGCTCGTAGTCCTGC-3´  R: 5´-AGGGAGTCGGGGGAAGAGAG-3´ | 912 | - | - | - |

PCR-RFLP, polymerase chain reaction-restriction fragment length polymorphism; SNP, single nucleotide polymorphism; F, forward; R, reverse;

^a^direct sequencing was applied for rs35678, rs 3774180, rs 3774179, and rs2278556.
