# Supplementary material for: The Evidence for Association of ATP2B2 Polymorphisms with Autism in Chinese Han Population
Source: PLoS One. 2013 Apr 19;8(4):e61021. doi: 10.1371/journal.pone.0061021 (PMC3631200; doi:10.1371/journal.pone.0061021)
Supplement: Table S3 — Information of 5 SNPs in ATP2B2 and the genotype frequencies in 427 autism trios of Chinese Han descent. (DOC) [file pone.0061021.s003.doc]

**Table S3. Information of 5 SNPs in *ATP2B2* and the genotype frequencies in 427 autism trios of Han Chinese descent.**

| Chr. position | Marker | Location | Genotype frequencies in children | | | *p* *_HWE_* ^a^ | Genotype frequencies in parents | | | *p* *_HWE_* ^b^ |
| --- | --- | --- | --- | --- | --- | --- | --- | --- | --- | --- |
| 10379923 | rs35678 | exon | CC | CT | TT | 0.770 | CC | CT | TT | 0.815 |
|  |  | (synonymous) | 75 | 199 | 140 |  | 148 | 402 | 264 |  |
| 10387059 | rs241509 | intron | AA | AC | CC | 0.038 | AA | AC | CC | 0.161 |
|  |  |  | 113 | 229 | 77 |  | 239 | 429 | 158 |  |
| 10396988 | rs3774180 | intron | CC | CT | TT | 0.605 | CC | CT | TT | 0.687 |
|  |  |  | 125 | 205 | 93 |  | 255 | 406 | 171 |  |
| 10397069 | rs3774179 | intron | CC | CT | TT | 0.318 | CC | CT | TT | 0.591 |
|  |  |  | 15 | 113 | 294 |  | 32 | 250 | 550 |  |
| 10402103 | rs2278556 | intron | AA | AG | GG | 0.061 | AA | AG | GG | 0.386 |
|  |  |  | 160 | 185 | 78 |  | 302 | 392 | 144 |  |

^a^ Hardy-Weinberg equilibrium *p* value for genotype distributions in children affected with autism; ^b^ Hardy-Weinberg equilibrium *p* value for genotype distributions in parents.
