# Supplementary material for: The Evidence for Association of ATP2B2 Polymorphisms with Autism in Chinese Han Population
Source: PLoS One. 2013 Apr 19;8(4):e61021. doi: 10.1371/journal.pone.0061021 (PMC3631200; doi:10.1371/journal.pone.0061021)
Supplement: Table S4 — Estimated haplotype frequencies and results of haplotype association analyses of ATP2B2 . (DOC) [file pone.0061021.s004.doc]

**Table S4. Estimated haplotype frequencies and results of haplotype association analyses of *ATP2B2*.**

| Haplotype | Freq | Families | S | E(S) | Var(S) | Z | *p* | Global | |
| --- | --- | --- | --- | --- | --- | --- | --- | --- | --- |
|  |  |  |  |  |  |  |  | χ^2^ | *p* |
| rs35678- rs241509 |  |  |  |  |  |  |  |  |  |
| T-A | 0.450 | 192.5 | 220.2 | 220.4 | 39.0 | -0.028 | 0.978 | 3.073 | 0.546 |
| C-C | 0.333 | 198.7 | 162.2 | 166.4 | 43.7 | -0.631 | 0.528 |  |  |
| T-C | 0.122 | 145.5 | 96.8 | 87.1 | 32.8 | 1.690 | 0.091 |  |  |
| C-A | 0.095 | 118.3 | 65.8 | 69.4 | 26.4 | -0.697 | 0.486 |  |  |
| rs35678- rs241509-3774180 |  |  |  |  |  |  |  |  |  |
| T-A-C | 0.417 | 189.4 | 210.3 | 212.6 | 39.8 | -0.360 | 0.719 | 3.943 | 0.414 |
| C-C-T | 0.318 | 203.1 | 162.6 | 165.1 | 45.1 | -0.381 | 0.703 |  |  |
| T-C-T | 0.100 | 120.3 | 80.2 | 69.9 | 28.2 | 1.929 | 0.054 |  |  |
| C-A-C | 0.090 | 107.6 | 60.4 | 63.9 | 24.6 | -0.718 | 0.473 |  |  |
| rs35678- rs241509-3774180-3774179 |  |  |  |  |  |  |  |  |  |
| C-C-T-T | 0.315 | 205.1 | 179.2 | 180.0 | 44.6 | -0.121 | 0.904 | 8.588 | 0.127 |
| T-A-C-T | 0.265 | 205.1 | 170.4 | 161.7 | 45.7 | 1.286 | 0.198 |  |  |
| T-A-C-C | 0.153 | 161.8 | 90.7 | 102.3 | 38.2 | -1.884 | 0.060 |  |  |
| T-C-T-T | 0.097 | 120.0 | 80.2 | 69.8 | 28.0 | 1.961 | 0.050 |  |  |
| C-A-C-T | 0.060 | 76.5 | 47.5 | 46.3 | 17.5 | 0.298 | 0.766 |  |  |
| rs35678- rs241509-3774180-3774179-rs2278556 |  |  |  |  |  |  |  |  |  |
| C-C-T-T-G | 0.253 | 190.6 | 153.7 | 155.7 | 43.2 | -0.306 | 0.759 | 6.266 | 0.394 |
| T-A-C-T-A | 0.252 | 196.5 | 165.7 | 161.2 | 43.9 | 0.685 | 0.493 |  |  |
| T-A-C-C-A | 0.149 | 159.0 | 88.2 | 101.0 | 37.7 | -2.086 | 0.037 |  |  |
| T-C-T-T-G | 0.086 | 109.8 | 67.7 | 63.1 | 25.6 | 0.914 | 0.361 |  |  |
| C-C-T-T-A | 0.061 | 77.5 | 41.4 | 43.4 | 18.9 | -0.456 | 0.648 |  |  |
| C-A-C-T-A | 0.059 | 74.7 | 45.6 | 45.5 | 16.9 | 0.034 | 0.973 |  |  |
| rs241509-rs3774180 |  |  |  |  |  |  |  |  |  |
| A-C | 0.509 | 144.8 | 202.8 | 201.8 | 33.8 | 0.166 | 0.868 | 1.418 | 0.492 |
| C-T | 0.417 | 193.9 | 180.8 | 174.1 | 44.3 | 1.010 | 0.313 |  |  |
| rs241509-rs3774180-rs3774179 |  |  |  |  |  |  |  |  |  |
| C-T-T | 0.412 | 201.3 | 215.1 | 206.2 | 43.0 | 1.354 | 0.176 | 6.313 | 0.097 |
| A-C-T | 0.325 | 191.8 | 185.4 | 176.3 | 44.0 | 1.373 | 0.170 |  |  |
| A-C-C | 0.183 | 183.9 | 104.4 | 118.0 | 43.7 | -2.054 | 0.040 |  |  |
| rs241509-rs3774180-rs3774179-rs2278556 |  |  |  |  |  |  |  |  |  |
| C-T-T-G | 0.339 | 204.1 | 187.0 | 185.3 | 47.0 | 0.257 | 0.797 | 6.025 | 0.197 |
| A-C-T-A | 0.312 | 189.8 | 187.5 | 182.0 | 43.1 | 0.824 | 0.410 |  |  |
| A-C-C-A | 0.180 | 182.5 | 102.0 | 117.2 | 43.4 | -2.306 | 0.021 |  |  |
| C-T-T-A | 0.073 | 95.1 | 58.3 | 53.9 | 23.1 | 0.902 | 0.367 |  |  |

Freq, estimated haplotype frequency; S, test statistics for the observed number of transmitted haplotypes; E(S), expected value of S under the null hypothesis (i.e., no linkage and no association).
