# Supplementary material for: The Evidence for Association of ATP2B2 Polymorphisms with Autism in Chinese Han Population
Source: PLoS One. 2013 Apr 19;8(4):e61021. doi: 10.1371/journal.pone.0061021 (PMC3631200; doi:10.1371/journal.pone.0061021)
Supplement: Table S5 — Comparison of the allele frequencies for the five SNPs in CHB and CEU from HapMap data. (DOC) [file pone.0061021.s005.doc]

**Table S5. Comparison of the allele frequencies for the five SNPs in CHB and CEU from HapMap data.**

| Population | rs35678 | rs241509 | rs3774180 | rs3774179 | rs2278556 |
| --- | --- | --- | --- | --- | --- |
|  | C T | A C | A G | A G | A G |
| CHB (CHB and JPT) | 0.537 0.463 | 0.558 ^a^ 0.442 ^a^ | 0.512 0.488 | 0.850^a^ 0.150^a^ | 0.560 0.440 |
| CEU | 0.553 0.447 | 0.500 0.500 | 0.628 0.372 | 0.858 0.142 | 0.376 0.624 |

Afreq, allele frequency; SNP, single nucleotide polymorphism; CHB, Han Chinese in Beijing, China; JPT, Japanese in Tokyo, Japan; CEU, Utah residents with Northern and Western European ancestry from the CEPH collection; ^a^ allele frequencies in CHB and JPT.
