# Supplementary material for: The Evidence for Association of ATP2B2 Polymorphisms with Autism in Chinese Han Population
Source: PLoS One. 2013 Apr 19;8(4):e61021. doi: 10.1371/journal.pone.0061021 (PMC3631200; doi:10.1371/journal.pone.0061021)
Supplement: Text S1 — Details for PCR-RFLP (polymerase chain reaction-restriction fragment length polymorphism) and direct sequencing. (DOC) [file pone.0061021.s006.doc]

**Text S1. Details for PCR-RFLP (polymerase chain reaction-restriction fragment length polymorphism) and direct sequencing**

The PCR amplification was performed in a 15 μl volume, containing 1.5μl PCR Buffer (TaKaRa), 200μM dNTPs, 0.3μM of each primer, 1U of Taq DNA polymerase and 40ng genomic DNA. The conditions used for PCR amplification were an initial denaturation phase at 94°C for 5 min, followed by 36 cycles at 94°C for 30 sec, annealing at 61-63.5°C for 30 sec, and extension at 72°C for 40 sec, followed by a final extension phase at 72°C for 7 min. A 15μl aliquot of the PCR product mixtures was completely digested with 5 units of restriction enzyme PpuMI for 10-12 hours at 37°C. Digestion products were visualized through ethidium bromide staining after electrophoresis in 2%-3% agarose gels. Gels were blindly read by two independent raters with discrepancies resolved by re-genotyping. As for direct sequencing, it was performed after cleaning the PCR product using a BigDye Terminator Cycle Sequencing Ready Reaction Kit with Ampli Taq DNA polymerase (PE Biosystem). The inner primers were used for the cycle-sequencing reaction, and the fragments were separated by electrophoresis on an ABI PRISM 377-96 DNA Sequencer (Applied Biosystem, Foster City, U.S.A).
